# Superiority of urgent vs early endoscopic hemostasis in patients with upper gastrointestinal bleeding with high-risk stigmata

**DOI:** 10.1093/gastro/goab042

**Published:** 2021-11-02

**Authors:** Masayasu Horibe, Eisuke Iwasaki, Juntaro Matsuzaki, Fateh Bazerbachi, Tetsuji Kaneko, Kazuhiro Minami, Seiichiro Fukuhara, Tatsuhiro Masaoka, Naoki Hosoe, Yuki Ogura, Shin Namiki, Yasuo Hosoda, Haruhiko Ogata, Takanori Kanai

**Affiliations:** 1 Division of Gastroenterology and Hepatology, Department of Internal Medicine, Keio University School of Medicine, Tokyo, Japan; 2 Division of Gastroenterology and Hepatology, Mayo Clinic, MN, USA; 3 Interventional Endoscopy Program, CentraCare, St Cloud Hospital, MN, USA; 4 Department of Clinical Trial, Tokyo Metropolitan Children’s Medical Center, Tokyo, Japan; 5 Teikyo Academic Research Center, Teikyo University, Tokyo, Japan; 6 Center for Diagnostic and Therapeutic Endoscopy, Keio University Hospital, Tokyo, Japan; 7 Department of Gastroenterology and Hepatology, Tokyo Metropolitan Tama Medical Center, Tokyo, Japan; 8 Division of Gastroenterology, Department of Internal Medicine, National Hospital Organization Saitama National Hospital, Saitama, Japan

**Keywords:** UGIB, urgent endoscopy, non-variceal bleeding, variceal bleeding, HARBINGER, GBS, upper gastrointestinal bleeding

## Abstract

**Background:**

Guidelines recommend that all patients with upper gastrointestinal bleeding (UGIB) undergo endoscopy within 24 h. It is unclear whether a subgroup may benefit from an urgent intervention. We aimed to evaluate the influence of endoscopic hemostasis and urgent endoscopy on mortality in UGIB patients with high-risk stigmata (HRS).

**Methods:**

Consecutive patients with suspected UGIB were enrolled in three Japanese hospitals with a policy to perform endoscopy within 24 h. The primary outcome was 30-day mortality. Endoscopic hemostasis and endoscopy timing (urgent, ≤6 h; early, >6 h) were evaluated in a regression model adjusting for age, systolic pressure, heart rate, hemoglobin, creatinine, and variceal bleeding in multivariate analysis. A propensity score of 1:1 matched sensitivity analysis was also performed.

**Results:**

HRS were present in 886 of 1966 patients, and 35 of 886 (3.95%) patients perished. Median urgent-endoscopy time (*n* = 769) was 3.0 h (interquartile range [IQR], 2.0–4.0 h) and early endoscopy (*n* = 117) was 12.0 h (IQR, 8.5–19.0 h). Successful endoscopic hemostasis and urgent endoscopy were significantly associated with reduced mortality in multivariable analysis (odds ratio [OR], 0.22; 95% confidence interval [CI], 0.09–0.52; *P* = 0.0006, and OR, 0.37; 95% CI, 0.16–0.87; *P* = 0.023, respectively). In a propensity-score-matched analysis of 115 pairs, adjusted comparisons showed significantly lower mortality of urgent vs early endoscopy (2.61% vs 7.83%, *P* < 0.001).

**Conclusions:**

A subgroup of UGIB patients, namely those harboring HRS, may benefit from endoscopic hemostasis and urgent endoscopy rather than early endoscopy in reducing mortality. Implementing triage scores that predict the presence of such lesions is important.

## Introduction 

Gastrointestinal bleeding is a major cause of hospital admissions, incurring significant economic burden to healthcare systems, accounting for >500,000 hospital admissions, 2.2 million days of hospitalization, and $5 billion in direct costs according to a 2018 report in the USA [1]. When upper gastrointestinal bleeding (UGIB) is suspected, endoscopy is undertaken to treat high-risk stigmata (HRS) [2–9]. The ability to predict HRS before endoscopy is a meaningful and important parameter in clinical decision-making because patients without HRS could be managed as outpatients and patients harboring HRS need an expedited endoscopy [10–12]. Thus we developed and validated the Horibe gAstRointestinal BleedING prEdiction scoRe (HARBINGER), which consists of only three variables to predict HRS and manage suspected UGIB [7, 13].

Although endoscopic hemostasis of HRS significantly reduced recurrent bleeding and the need for urgent intervention and surgery, it is unclear whether performing endoscopic hemostasis leads to reduced mortality [14]. Furthermore, although guidelines recommend that all patients with UGIB undergo endoscopy within 24 h, the optimal timing of endoscopy for patients with HRS remains unclear [15].

In this study of patients suspected of having UGIB, we aimed to evaluate the influence of endoscopic hemostasis on overall mortality and identify the optimal timing of endoscopy in patients with HRS.

## Patients and methods

### Study design and patients

All consecutive adult patients with a suspected UGIB were enrolled at three acute care hospitals in Japan (Tama Medical Center between 2008 and 2015, and Keio University Hospital and Saitama National Hospital between 2012 and 2015). Although all clinically suspected cases of UGIB were included (i.e. presence of hematemesis, coffee-ground emesis, nasogastric lavage with blood or coffee-ground material, melena, tarry stools or black stools as determined by a rectal examination or medical history), we excluded patients with post-procedural UGIB (e.g. post endoscopic submucosal dissection). The institutional review board of each hospital approved the study (20130069). Informed consent or an opt-out clause was obtained from each patient.

### Outcome and clinical details

The primary outcome was the all-cause mortality within 30 days after a suspected UGIB. We divided patients into two groups: those found to have HRS on endoscopy and those without HRS. One of the following conditions was defined as HRS: (i) when the source of bleeding was peptic ulcer disease, spurting, gushing, oozing bleeding, or non-bleeding visible vessel (Ia, Ib, or IIa in the Forrest classification) was seen [4]; (ii) when the source of bleeding was variceal hemorrhage, active bleeding, or evidence of recent bleeding (e.g. red or white plug) was found; (iii) when other sources of bleeding, not related to peptic ulcer disease or variceal hemorrhage, were identified, we considered HRS to be spurting or gushing bleeding while oozing lesions, which resolved spontaneously, were not deemed as such. The evaluation was done after removing an adherent clot if one was encountered.

The determination of HRS was adjudicated by showing the endoscopic images of all encountered lesions to experienced endoscopists who were blind to the clinical information of patients. The cause of death, when it occurred, was captured in the medical record.

To achieve endoscopic hemostasis, one of or a combination of the following techniques was implemented: argon plasma coagulation, electrocautery, clips, band ligation, epinephrine injections, or sclerosants injection. An urgent endoscopy was defined as one performed within 6 h after the presentation of suspected UGIB, while early endoscopy was done >6 hours of presentation [16, 17]. We also calculated the HARBINGER and Glasgow Blatchford Score (GBS) at the time of presentation [7, 13, 18].

### Statistical analysis

Categorical variables are expressed in number and percentage, and were compared using the chi-square test or Fisher's exact test. Quantitative numerical variables are presented by mean and standard deviation (SD) or median and interquartile range (IQR), and were compared using Student’s *t*-test or the Mann–Whitney *U* test. The best cut-off value of endoscopy timing for mortality was calculated by a receiver-operating characteristic (ROC) curve with the greatest sensitivity −(1− specificity). The odds ratio (OR) and 95% confidence interval (CI) of endoscopic hemostasis and endoscopic timing were calculated using univariable and multivariable logistic-regression analysis.

Three multivariable logistic-regression models with different confounding factors were used to demonstrate robustness because the number to be adjusted for as a confounding factor was limited due to the small number of mortality events (*n* = 50). The continuous variables in confounding factors were classified into binary categorical variables according to the existing cut-off in the literature or ROC. It means that we used the cut-off of AIMS65 for age (≥65 years), the cut-offs of GBS for systolic blood pressure (SBP) (<90 mmHg) and heart rate (HR) (≥100 beats/min), and the cut-off of the randomized–controlled trial (RCT) in the *New England Journal of Medicine* for hemoglobin level (<7.0 g/dL), but the cut-offs for creatinine (≥1.0 mg/dL) were not available in the past literature, so we calculated the optimal cut-off from our current data [18–20]. The confounding factors in model 1 were chosen as those that were significantly different in both mortality and urgent-endoscopy comparisons (Tables 1 and 2): age, SBP, HR, index presentation hemoglobin level, creatinine, and variceal bleeding. The confounding factors in model 2 were chosen based on the literature and each study site: age, gender, congestive heart failure, any hepatic disease, variceal bleeding, and institution (Tama, Keio, and Saitama). The confounding factors in model 3 were other treatments including chosen proton-pump-inhibitors use, blood transfusion, and variceal bleeding.

**Table 1.** Differences in characteristics and treatments between patients who survived and those who perished

	No. of individuals	Alive (*n* = 1,916)	Dead (*n* = 50)	*P*-value
Characteristic				
Age, mean (SD), years	1,916/50	68.4 (15.6)	73.0 (12.0)	0.041^a^
Male, *n* (%)	1,916/50	1,271 (66.3)	36 (72.0)	0.45^b^
Any hepatic disease, *n* (%)	1,916/50	271 (14.1)	12 (24.0)	0.06^b^
Congestive heart failure, *n* (%)	1,916/50	122 (6.37)	5 (10.0)	0.25^b^
SBP, mean (SD), mmHg	1,907/50	113 (26.6)	92 (30.6)	<0.0001^a^
HR, mean (SD), beats/min	1,905/50	92.6 (20.5)	99.9 (22.3)	0.013^a^
Hemoglobin, mean (SD), g/dL	1,904/50	9.2 (2.9)	8.3 (2.7)	0.039^a^
BUN, mean (SD), mg/dL	1,904/50	37.8 (25.4)	44.8 (24.8)	0.061^a^
Creatinine, median (IQR), mg/dL	1,903/50	0.87 (0.68–1.3)	1.2 (0.80–1.9)	0.003^c^
Endoscopic timing, median (IQR), h	1,916/50	4.0 (2.0–6.0)	3.5 (2.0–7.3)	0.77^c^
Variceal bleeding, *n* (%)	1,916/50	174 (9.1)	13 (26.0)	<0.0001^b^
HRS, *n* (%)	1,916/50	851 (44.4)	35 (70.0)	0.0003^b^
Treatment				
Use of PPI, *n* (%)	1,916/50	1,652 (86.2)	48 (96.0)	0.056^b^
Blood transfusion, *n* (%)	1,916/50	1,036 (54.1)	43 (86.0)	<0.0001^c^
Endoscopic hemostasis, *n* (%)	1,916/50	857 (44.7)	27 (54)	0.20^c^
Interventional radiology, *n* (%)	1,916/50	19 (0.99)	3 (6.00)	0.017^d^
Surgery, *n* (%)	1,916/50	13 (0.68)	1 (2.00)	0.30^d^

BUN, blood urea nitrogen; HR, heart rate; HRS, high-risk stigmata; IQR, interquartile range; PPI, proton-pump inhibitors; SBP, systolic blood pressure; SD, standard division.

^a^ Student’s *t*-test.

^b^ Chi-square test.

^c^ Mann–Whitney *U* test.

^d^ Fisher's exact test.

**Table 2.** Differences in characteristics, treatments, and outcomes of patients who underwent urgent vs early endoscopy

	No. of individuals	Urgent endoscopy (*n* = 1,490)	Early endoscopy (*n* = 476)	*P*-value
Characteristic				
Age, mean (SD), years	1490/476	68.1 (15.6)	69.8 (15.5)	0.039^a^
Male, *n* (%)	1490/476	1,015 (68.1)	292 (61.3)	0.0064^b^
Any hepatic disease, *n* (%)	1490/476	225 (15.1)	58 (12.2)	0.11^b^
Congestive heart failure, *n* (%)	1490/476	86 (5.8)	41 (8.6)	0.028^b^
SBP, mean (SD), mmHg	1488/469	111 (27.0)	117 (25.9)	<0.001^a^
HR, mean (SD), beats/min	1485/470	94.1 (20.9)	88.7 (19.0)	<0.001^a^
Hemoglobin, mean (SD), g/dL	1488/466	9.1 (2.7)	9.4 (3.0)	0.017^a^
BUN, mean (SD), mg/dL	1487/466	39.5 (25.5)	33.3 (25.4)	<0.0001^a^
Creatinine, median (IQR), mg/dL	1487/466	0.88 (0.69–1.3)	0.86 (0.65–1.1)	0.016^c^
Variceal bleeding, *n* (%)	1490/476	166 (11.1)	21 (4.4)	<0.0001^b^
HRS, *n* (%)	1490/476	769 (51.6)	117 (24.6)	<0.0001^b^
Treatment, *n* (%)				
Use of PPI	1490/476	1,327 (89.1)	373 (78.4)	<0.0001^b^
Blood transfusion	1490/476	859 (57.7)	220 (46.2)	<0.0001^b^
Endoscopic hemostasis	1490/476	762 (51.1)	122 (25.6)	<0.0001^b^
Interventional radiology	1490/476	16 (1.07)	6 (1.26)	0.74^b^
Surgery	1490/476	11 (0.74)	3 (0.63)	1.00^d^
Outcome, *n* (%)				
All-cause mortality	1490/476	37 (2.48)	13 (2.73)	0.76^b^
Death due to UGIB	1490/476	12 (0.81)	7 (1.47)	0.20^b^
Death unrelated to UGIB	1490/476	25 (1.68)	6 (1.26)	0.52^b^

BUN, blood urea nitrogen; HR, heart rate; HRS, high-risk stigmata; IQR, interquartile range; PPI, proton-pump inhibitors; SBP, systolic blood pressure; SD, standard division; UGIB, upper gastrointestinal bleeding.

^a^ Student’s *t*-test.

^b^ Chi-square test.

^C^ Mann–Whitney *U* test.

^d^ Fisher's exact test.

Since pursuing endoscopy ≤6 or >6 h was not randomly assigned, propensity-score matching was applied to minimize possible confounding and selection biases between the urgent (≤6 h) and early (>6 h) endoscopy groups in patients with HRS [21]. Propensity scores, which have the advantage that there is no limit to the number of confounding factors that need to be accounted for, were calculated with multivariable logistic regression using all the characteristics that could be obtained before performing the endoscopy. The propensity-score model included age, gender, any hepatic disease, congestive heart failure, SBP, HR, index presentation hemoglobin level, blood urea nitrogen, and creatinine. We formed matched pairs between urgent- and early-endoscopy groups using a one-to-one nearest neighbor approach with a caliper width of 0.2 without replacement. McNemar test was performed to evaluate the efficacy of urgent endoscopy [22].

As sensitivity analyses, time cut-offs (≤6 vs 6–12 vs >12 h) [16, 23, 24], subgroup analysis (variceal-bleeding group and non-variceal-bleeding group with HRS) and threshold analyses (HARBINGER ≥2, GBS ≥12 and SBP <90 mmHg [hemodynamic instability] groups) for UGIB-specific mortality were evaluated [7, 13, 25]. A two-sided *p*-value <0.05 was considered statistically significant. All analyses were done using JMP(r) statistical software version 14.1.0 (SAS Institute, Inc., Cary, NC, USA).

## Results

**Figure 1.**
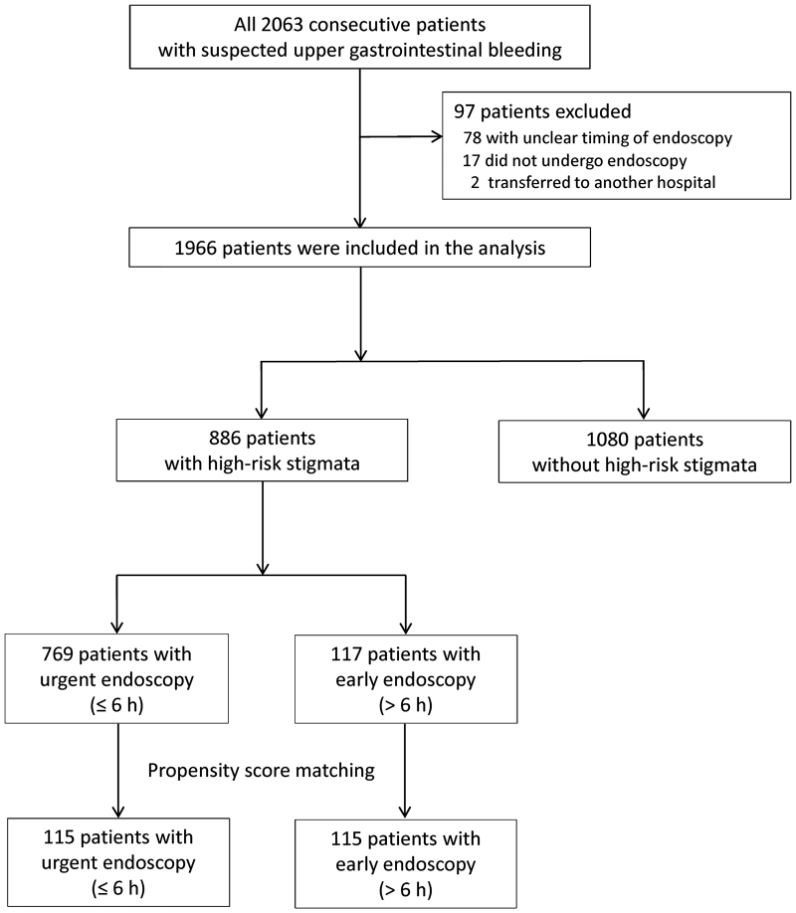
Flow diagram of patients recruited into the study

The study flow is shown in Figure 1. Of 2,063 consecutive patients with suspected UGIB in three institutions, 97 patients were excluded and reasons for exclusion are provided in Figure 1. The remaining 1,966 patients were included in the analysis; the mean age in the cohort was 68.6 years (SD, 15.5 years) and 1,307 (66.5%) were males. The etiologies of UGIB are shown in Table 3. The median duration from the first clinical examination to the initiation of endoscopy was 3.8 h (IQR, 2.0–6.0 h). Fifty patients perished (2.5%) within 30 days from presentation, of which 19 deaths (1.0%) were related to UGIB. Table 1 shows the differences in characteristics between patients who survived and those who perished. Table 2 shows differences in characteristics, treatments, and outcomes of patients who underwent urgent vs early endoscopy.

**Table 3.** The etiologies of upper gastrointestinal bleeding

Diagnosis	*n* (%)
Peptic ulcer	993 (50.5)
Varix	187 (9.5)
Neoplasia	103 (5.2)
Mallory–Weiss syndrome	99 (5.0)
Erosion	212 (10.8)
Normal	171 (8.7)
Other	201 (10.2)

### HRS group

Among 1,966 patients, 886 had HRS, of whom 35 (3.95%) patients perished (19 events were related to bleeding and 16 to other causes). In this group of patients with HRS, the relationship between endoscopic timing and mortality was assessed, and the best cut-off value to achieve the lowest mortality was 5.5 h. The median time of urgent endoscopy (≤6 h) group was 3.0 h (IQR, 2.0–4.0 h) in 769 patients and that of the early endoscopy (>6 h) group was 12.0 h (IQR, 8.5–19.0 h) in 117 patients. Endoscopic hemostasis and urgent endoscopy were significantly associated with lower mortality in univariable analysis (Table 4). After adjusting for confounding factors in three models of multivariable analysis, both endoscopic hemostasis and urgent endoscopy were significantly associated with lower overall mortality (Table 4).

**Table 4.** Factors influencing overall mortality in suspected UGIB patients with HRS and those without HRS

Factor	Univariable analysis		Multivariable analysis					
			Model 1		Model 2		Model 3	
	OR (95% CI)	*P*-value	Adjusted OR (95% CI)	*P*-value	Adjusted OR (95% CI)	*P*-value	Adjusted OR (95% CI)	*P*-value
HRS group								
Endoscopic hemostasis	0.21 (0.10–0.49)	0.0007	0.22 (0.09–0.52)	0.0006	0.20 (0.08–0.46)	0.0002	0.18 (0.08–0.43)	<0.0001
Endoscopy within 6 h	0.42 (0.20–0.97)	0.043	0.37 (0.16–0.87)	0.023	0.37 (0.15–0.88)	0.024	0.40 (0.18–0.93)	0.034
Non-HRS group								
Endoscopic hemostasis	1.13 (0.15–8.78)	0.90	1.09 (0.14–8.66)	0.90	0.84 (0.10–6.75)	0.87	0.98 (0.13–7.66)	0.98
Endoscopy within 6 h	1.38 (0.47–4.99)	0.58	1.05 (0.32–3.41)	0.93	1.39 (0.40–4.79)	0.61	1.16 (0.36–3.74)	0.80

CI, confidence interval; HRS, high-risk stigmata; OR, odds ratio; UGIB, upper gastrointestinal bleeding.

Model 1 includes variables: age (≥65 years), systolic blood pressure (<90 mmHg), heart rate (≥100 beats/min), index presentation hemoglobin level (<7.0 g/dl), creatinine (≥1.0 mg/dL), and variceal bleeding.

Model 2 includes variables: age (≥65 years), gender (male), congestive heart failure, any hepatic disease, variceal bleeding, and study site (Tama, Keio, and Saitama).

Model 3 includes variables: proton-pump-inhibitors use, blood transfusion, and variceal bleeding.

After propensity-score matching, 115 matched pairs were generated from 769 patients in the urgent-endoscopy group and 117 patients in the early-endoscopy group in a 1:1 manner. There were no significant differences in characteristics between the two matched groups (Table 5). The mortality in the urgent-endoscopy group was significantly lower than that in the early-endoscopy group (2.61% vs 7.83%, *P* < 0.001).

**Table 5.** Comparison of characteristics and outcome of the urgent- and early-endoscopy groups after propensity matching in patients with high-risk stigmata

Characteristics	Urgent endoscopy (*n* = 115)	Early endoscopy (*n* = 115)	*P*-value
Age, mean (SD), years	71.4 (12.1)	70.2 (12.3)	0.47^a^
Male, *n* (%)	75 (65.2)	72 (62.6)	0.68^b^
Any hepatic disease, *n* (%)	30 (26.1)	21 (18.3)	0.15^b^
Congestive heart failure, proton-pump *n* (%)	4 (3.5)	6 (5.2)	0.74^c^
SBP, mean (SD), mmHg	106 (28.2)	104 (25.8)	0.49^a^
HR, mean (SD), beats/min	95.4 (20.2)	94.6 (18.0)	0.74^a^
Hemoglobin, mean (SD), g/L	8.9 (2.7)	8.9 (2.5)	0.97^a^
BUN, mean (SD), mg/dL	40.4 (21.7)	41.2 (30.3)	0.81^a^
Creatinine, median (IQR), mg/dL	0.82 (0.63–1.22)	0.79 (0.61–1.10)	0.48^d^
All-cause mortality, *n* (%)	3 (2.61)	9 (7.83)	<0.001^e^

BUN, blood urea nitrogen; HR, heart rate; IQR, interquartile range; SBP, systolic blood pressure; SD, standard deviation.

^a^ Student’s *t*-test.

^b^ Chi-square test.

^c^ Fisher's exact test.

^d^ Mann–Whitney *U* test.

^e^ McNemar test.

**Figure 2.**
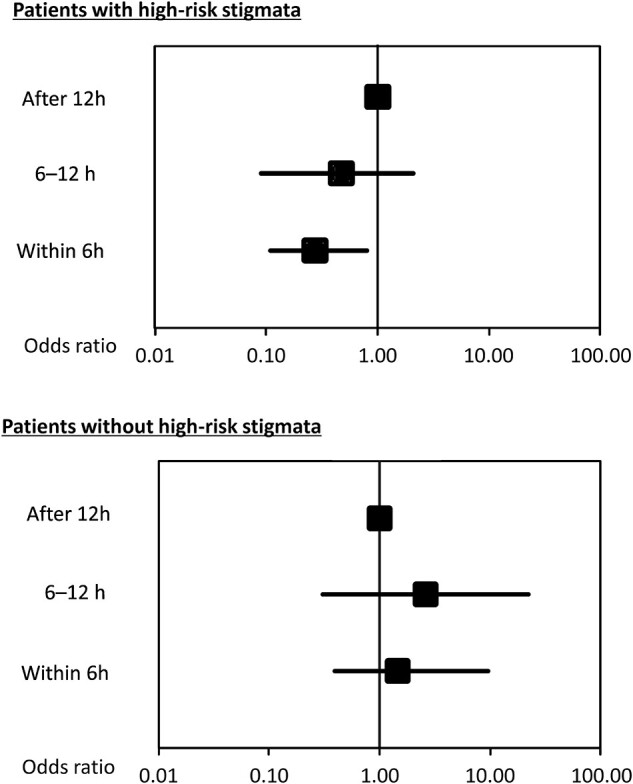
Comparison of overall mortality in three subgroups stratified by high-risk stigmata (HRS) and non-HRS

We also compared three groups (≤6, 6–12, and >12 h). Endoscopy ≤6 h was significantly associated with lower mortality than endoscopy >12 h in univariable analysis (OR, 0.29; 95% CI, 0.12–0.81; *P* = 0.021), while endoscopy 6–12 h was not (OR, 0.68; 95% CI, 0.23–2.90; *P* = 0.55). In multivariable logistic analysis (model 1), endoscopy ≤6 h was significantly associated with lower mortality (OR, 0.27; 95% CI, 0.11–0.80; *P* = 0.020) compared with endoscopy >12 h, while there was no significant difference in mortality between two groups (6–12 and >12 h groups) (OR, 0.47; 95% CI, 0.09–2.09; *P* = 0.33) (Figure 2).

### Non-HRS group

In the non-HRS group of 1,080 patients, mortality in 15 patients (1.39%) was not related to bleeding, but was due to other causes (e.g. end-stage malignancy, infection, organ failure). Neither endoscopic hemostasis nor urgent endoscopy was significantly associated with reduced mortality in both univariable and three models of multivariable logistic analysis (Table 4). Among three groups (endoscopy ≤6, 6–12, and >12 h), endoscopy ≤6 h, and 6–12 h were not significantly associated with mortality in univariable analysis (OR, 1.99; 95% CI, 0.53–12.9; *P* = 0.34, and OR, 2.62; 95% CI, 0.31–22.1; *P* = 0.35, respectively). In the multivariable logistic analysis, endoscopy ≤6 and 6–12 h compared with >12 h were not significantly associated with mortality (OR, 1.35; 95% CI, 0.34–9.0; *P* = 0.70, and OR, 2.16; 95% CI, 0.25–18.7; *P* = 0.46, respectively) (Figure 2).

### Sensitivity analyses

#### Influence of urgent endoscopy in variceal-bleeding and non-variceal-bleeding-with-HRS groups

Of 886 patients with HRS, 143 patients had variceal bleeding and 743 had non-variceal bleeding (Table 6). In the variceal-bleeding-with-HRS group, urgent endoscopy was not significantly associated with overall mortality (OR, 0.50; 95% CI, 0.11–3.52; *P* = 0.44), while in the non-variceal-bleeding-with-HRS group, urgent endoscopy was significantly associated with lower overall mortality (OR, 0.35; 95% CI, 0.14–0.88; *P* = 0.025).

**Table 6.** Comparison of mortality in urgent- and early-endoscopy groups in patients with HRS divided into variceal and non-variceal bleeding

Characteristic	Urgent endoscopy	Early endoscopy	OR (95% CI)	*P*-value
Variceal-bleeding patients with HRS (*n* = 143)	7.8% (10/129)	14.3% (2/14)	0.50 (0.11–3.52)	0.44
Non-variceal-bleeding patients with HRS (*n* = 743)	2.5% (16/640)	6.8% (7/103)	0.35 (0.14–0.88)	0.025

CI, confidence interval; HRS, high-risk stigmata; OR, odds ratio.

#### Influence of urgent endoscopy stratified by HARBINGER ≥2, GBS ≥12, and SBP <90 mmHg (hemodynamic instability) groups

Of 1,108 patients with HARBINGER ≥2, HRS was found in 735 (66.3%). After adjusting for confounders (model 1) in multivariable logistic analysis (Table 7), although urgent endoscopy was not significantly associated with overall mortality (OR, 0.56; 95% CI, 0.24–1.30; *P* = 0.18), it was significantly associated with decreased UGIB-specific mortality in multivariable logistic-regression analysis (OR, 0.30; 95% CI, 0.09–0.94; *P* = 0.04) (Table 8).

**Table 7.** Components of HARBINGER [7, 13] and GBS [18] scoring systems

HARBINGER		GBS	
Range, 0–3	Point	Range, 0–23	Point
Absence of daily PPI use in the preceding week	1	Urea, mmol/L	
Shock index ≥1 (HR/SBP ≥1)	1	≥6.5 to <8.0	2
BUN/Cr ≥30 (Urea/Cr ≥140)	1	≥8.0 to <10.0	3
		≥10.0 to <25.0	4
		≥25.0	6
		Hemoglobin, g/L, for men	
		≥120 to <130	1
		≥100 to <120	3
		<100	6
		Hemoglobin, g/L, for women	
		≥100 to <120	1
		<100	6
		SBP, mmHg	
		100–109	1
		90–99	2
		<90	3
		Other makers	
		HR ≥100 beats/min	1
		Melena	1
		Syncope	2
		Hepatic disease	2
		Cardiac failure	2

HARBINGER, Horibe gAstRointestinal BleedING prEdiction scoRe; GBS, Glasgow Blatchford Score; BUN, blood urea nitrogen; Cr, creatinine; HR, heart rate; PPI, proton-pump inhibitor; SBP, systolic blood pressure.

**Table 8.** The adjusted odds ratio of urgent endoscopy for mortality and UGIB-specific mortality in subgroup patients^a^

Factor	HARBINGER ≥2, *n* = 1,108 (HRS: 66.3%)		GBS ≥12, *n *= 730 (HRS: 58.5%)		SBP <90 mmHg, *n* = 389 (HRS: 71.7%)	
	Adjusted OR (95% CI)	*p*-value	Adjusted OR (95% CI)^a^	*p*-value	Adjusted OR (95% CI)^a^	*p*-value
Mortality						
Endoscopy within 6 h	0.56 (0.24–1.30)	0.18	0.80 (0.29–2.22)	0.67	0.69 (0.24–2.00)	0.50
UGIB-specific mortality						
Endoscopy within 6 h	0.30 (0.09–0.94)	0.04	0.43 (0.08–2.32)	0.33	0.31 (0.08–1.15)	0.08

CI, confidence interval; GBS, Glasgow Blatchford Score; HARBINGER, Horibe gAstRointestinal BleedING prEdiction scoRe; HRS, high-risk stigmata; OR, odds ratio; SBP, systolic blood pressure; UGIB, upper gastrointestinal bleeding.

^a^ Multivariable logistic analysis includes variables: age (≥65 years), SBP (<90 mmHg), heart rate (≥100 beats/min), index presentation hemoglobin level (<7.0 g/dl), creatinine (≥1.0 mg/dL), and variceal bleeding.

Of 730 patients with GBS ≥12, HRS was found in 427 (58.5%). After adjusting for confounders (model 1) in multivariable logistic analysis, urgent endoscopy was not significantly associated with mortality and UGIB-specific mortality.

Of 389 patients with SBP <90 mmHg (hemodynamic instability), HRS was found in 279 (71.7%). After adjusting for confounders (model 1) in multivariable logistic analysis, urgent endoscopy was not significantly associated with mortality and UGIB-specific mortality.

## Discussion

In this large multicenter cohort of Japanese patients with suspected UGIB, we found that performing endoscopic hemostasis and urgent endoscopy (≤6 h from presentation) were factors associated with lower overall mortality compared with early endoscopy (>6 h), but only in those patients later found to harbor HRS. In contrast, those patients without HRS did not benefit from this urgent intervention. In addition, the mortality rate was significantly lower with urgent endoscopy either in multivariate logistic-regression analysis or in the propensity-score-matched analysis. Although the *P*-value of the propensity-matching score is 0.047, the results are likely robust and unlikely attributed to chance because all analyses showed a significant difference in the influence of urgent endoscopy significantly reducing mortality. In a subgroup analysis, urgent endoscopy was significantly associated with lower mortality rates in patients with non-variceal bleeding. Although urgent endoscopy has been recommended for patients with variceal bleeding with HRS, this result may support that the patients with non-variceal bleeding may also benefit from urgent endoscopy when HRS are suspected to be present [5].

HRS are defined by well-established endoscopic findings indicating the need for endoscopic hemostasis by international consensus statements [2–9]. To the best of our knowledge, although improved outcomes, other than death, were reported in patients with HRS, this is the first study to show an association between endoscopic hemostasis and decreased all-cause mortality [14]. When contrasted with previous studies, our findings may be different due to the relatively short interval in conducting endoscopy in the urgent group (median 3.5 h) as well as the adjudication of HRS by endoscopy experts blinded to the clinical condition of the patient.

To date, there are no RCTs designed to randomly allocate endoscopy timing in UGIB patients based on their likelihood of harboring HRS [26]. Nonetheless, in certain high-risk groups (e.g. GBS >7, patients with frankly bloody nasogastric output), one observational study and one RCT showed that earlier endoscopy (≤6 vs 6–48 h and ≤12 vs >12 h, respectively) was significantly associated with better outcomes [16, 23]. On the other hand, a large cohort study suggested that urgent endoscopy ≤6 h in hemodynamically unstable patients may worsen mortality [27] and a recent RCT showed no benefit from urgent endoscopy ≤6 h in a high-risk population (GBS ≥12) of patients with UGIB [17]. These discrepancies between studies may be due to the variability in the targeted high-risk groups. For example, the GBS was designed to predict composite outcomes (e.g. need for intervention/transfusion, mortality) and was not intended to predict the presence of HRS. Indeed, of the enrolled 516 patients with GBS ≥12, 280 (54.3%) required endoscopic treatment [17]. It is reasonable to deduce that urgent endoscopy could not improve the prognosis in about half of patients with GBS ≥12 who did not require endoscopic hemostasis. In this context, in our current study, urgent endoscopy ≤6 h was not associated with a statistically significant improvement in all-cause mortality or UGIB mortality. We surmise that more cases may be needed to detect a significant difference in mortality for urgent endoscopy when choosing GBS ≥12 as a high-risk group of interest.

The HARBINGER, which has been recently validated, is the first simple score incepted to predict the presence of HRS [7, 13]. The HARBINGER (AUC = 0.78) had significantly high accuracy of predicting HRS compared with the GBS (AUC = 0.68) [13]. In our current study, patients with HARBINGER ≥2 included 66.3% (735/1108) patients with HRS and those had benefited from urgent endoscopy ≤6 h in reducing UGIB-specific mortality compared with early endoscopy (>6 h), albeit not from an all-cause-mortality standpoint. This finding may support that patients with HARBINGER ≥2 should receive urgent endoscopy [7, 13]. However, HRS were found in 58.5% (427/730) of patients with GBS ≥12 and they had not benefited from urgent endoscopy in reducing UGIB-specific mortality and all-cause mortality. Moreover, the HARBINGER ≥2 (56.4%, 1108/1966) accounted for a higher proportion among patients with suspected UGIB compared with GBS ≥12 (37.1%, 730/1966). Thus, the HARBINGER may be a better stratification tool for RCTs than GBS when patients are allocated to undergo urgent endoscopy.

Our study has several shortcomings, inherent in the lack of randomization and the restriction of our demographics to Japanese patients. To ascertain whether mortality is truly reduced as a result of urgent intervention in patients with HRS, a powered RCT is needed in which patients are randomized according to the HARBINGER. However, ethical considerations may not permit the randomization of such high-risk groups.

In addition, it is almost certain that there are other confounders not controlled for our analyses. For example, we were unable to calculate for the American Society of Anesthesiologists score for each patient as underlying conditions and the size/number of ulcers was not described in our database. We attempted to compensate for such shortcomings by adjusting and controlling for not only the confounding factors identified from the analysis of this study data, but also those identified in the literature, including congestive heart failure and any hepatic disease as important underlying diseases in UGIB and by other offered treatments (e.g. use of proton-pump inhibitors, blood transfusions). Further, we were able to describe the etiology of UGIB (Table 4) accurately. All sensitivity analyses were consistent in showing the benefit of urgent endoscopy and endoscopic hemostasis after adjusting for confounding factors including variceal bleeding. In the variceal-bleeding group, urgent endoscopy showed a trend toward lower mortality but did not differ significantly, which likely relates to the small sample size.

In conclusion, both endoscopic hemostasis and urgent endoscopy within 6 h from the presentation were significantly associated with lower all-cause mortality in patients who harbored HRS compared with early endoscopy (conducted >6 h) in a large multicenter Japanese cohort. In patients with HARBINGER ≥2, urgent endoscopy was significantly associated with reduced mortality from UGIB. Future studies should investigate whether UGIB patients’ triage, according to the HARBINGER, will accrue a mortality benefit when urgent endoscopic hemostasis is pursued.

## Authors’ Contributions

M.H., E.I., Te.K., J.M, T.M, N.H., Y.O., S.N., Y.H., H.O., and Ta.K. conceived of and designed the project; M.H., E.I., F.B., Te.K., and J.M analysed and interpreted the data; M.H., E.I., F.B., and J.M drafted the manuscript; M.H., E.I., F.B., Te.K., J.M, K.M, S.F., T.M, N.H., and Ta.K. made critical revisions to important intellectual content. All authors read and approved the final manuscript.

## Funding

None.
